# The lncRNA H19 binding to let‐7b promotes hippocampal glial cell activation and epileptic seizures by targeting Stat3 in a rat model of temporal lobe epilepsy

**DOI:** 10.1111/cpr.12856

**Published:** 2020-07-10

**Authors:** Chun‐Lei Han, Yun‐Peng Liu, Chen‐Jia Guo, Ting‐Ting Du, Ying Jiang, Kai‐Liang Wang, Xiao‐Qiu Shao, Fan‐Gang Meng, Jian‐Guo Zhang

**Affiliations:** ^1^ Department of Functional Neurosurgery Beijing Neurosurgical Institute Capital Medical University Beijing China; ^2^ Beijing Key Laboratory of Neurostimulation Beijing China; ^3^ Department of Neurosurgery Beijing Tiantan Hospital Capital Medical University Beijing China; ^4^ Department of Pathology School of Basic Medical Sciences Capital Medical University Beijing China; ^5^ Department of Neurology Beijing Tiantan Hospital Capital Medical University Beijing China

**Keywords:** astrocytes, inflammatory, lncRNA, microglia, microRNA, temporal lobe epilepsy

## Abstract

**Objectives:**

Glial cell activation contributes to the inflammatory response and occurrence of epilepsy. Our preliminary study demonstrated that the long non‐coding RNA, H19, promotes hippocampal glial cell activation during epileptogenesis. However, the precise mechanisms underlying this effect remain unclear.

**Materials and methods:**

H19 and let‐7b were overexpressed or silenced using an adeno‐associated viral vector in vivo. Their expression in a kainic acid‐induced epilepsy model was evaluated by real‐time quantitative PCR, fluorescence in situ hybridization, and cytoplasmic and nuclear RNA isolation. A dual‐luciferase reporter assay was used to evaluate the direct binding of let‐7b to its target genes and H19. Western blot, video camera monitoring and Morris water maze were performed to confirm the role of H19 and let7b on epileptogenesis.

**Results:**

H19 was increased in rat hippocampus neurons after status epilepticus, which might be due to epileptic seizure‐induced hypoxia. Increased H19 aggravated the epileptic seizures, memory impairment and mossy fibre sprouting of the epileptic rats. H19 could competitively bind to let‐7b to suppress its expression. Overexpression of let‐7b inhibited hippocampal glial cell activation, inflammatory response and epileptic seizures by targeting Stat3. Moreover, overexpressed H19 reversed the inhibitory effect of let‐7b on glial cell activation.

**Conclusions:**

LncRNA H19 could competitively bind to let‐7b to promote hippocampal glial cell activation and epileptic seizures by targeting Stat3 in a rat model of temporal lobe epilepsy.

## 1 INTRODUCTION

Temporal lobe epilepsy (TLE) is the most common form of epilepsy in adults; however, antiepileptic drugs are ineffective in one‐third of patients.^1^ Therefore, there is a need for a deeper understanding of the mechanisms underlying TLE. Recent studies show that postictal hypoxia is an important component of seizure‐induced brain damage.^2^ Hippocampal sclerosis induced by brain damage is characterized by glial cell activation and neuron loss, which is the hallmark of TLE pathology.^3^ Moreover, glial cell activation and proliferation can alter blood‐brain barrier integrity, iron and neurotransmitter homeostasis, cause an inflammatory response, resulting in neuronal hyperexcitability, as well as the generation and spread of seizure activity.^4^ However, the precise molecular mechanisms of glial cell activation remain unclear.

The long non‐coding RNA (lncRNA), H19, is one of the first identified lncRNAs.^5^ Despite being identified several decades ago, the function of H19 is poorly understood and its involvement in pathological conditions has only recently begun to be investigated.^6^ Recent studies demonstrate that H19 is upregulated in hypoxic stress, and furthermore, it possesses oncogenic properties.^7^, ^8^ It has also been shown that H19 can regulate gene expression by acting as a competing endogenous RNA (ceRNA) to sponge let‐7.^9^, ^10^, ^11^, ^12^, ^13^ The let‐7 is the first known human miRNA,^14^ and both the sequence and function of mature let‐7 are highly conserved across species. In addition, let‐7 plays an important role in cell development and maturation.^15^ Let‐7b is a member of the let‐7 family that has been shown to play a role as a tumour suppressor in tumour‐related diseases.^16^, ^17^ In addition to cancerous diseases, let‐7b has also been shown to inhibit neural stem cell proliferation, accelerate neural differentiation^15^ and protect mesenchymal stem cells from apoptosis and autophagy following implantation into infarcted myocardium.^18^ Furthermore, let‐7b has been used as an adjunct to improve the diagnostic performance of biomarkers for Alzheimer's disease.^19^

Our previous work demonstrated that H19 contributed to hippocampal glial cell activation in a rat model of TLE.^20^ We also found that H19 displayed a strong negative correlation with let‐7b (let‐7b‐5p) expression during epileptogenesis.^21^, ^22^ Therefore, we speculate that H19 might promote hippocampal glial cell activation by targeting let‐7b during the epileptogenesis as a ceRNA. In the present study, we intend to explore the detailed function and mechanism of H19 and let‐7b in epileptogenesis.

## 2 MATERIALS AND METHODS

### 2.1 Animals

Male Sprague‐Dawley rats (200‐220 g body weight) were obtained from Vital‐River Experimental Animal Technology, Co., Ltd. The animals were housed in a temperature‐controlled room under a standard 12‐hours light/dark cycle and supplied with free access to standard rodent chow and water. All animal experimental procedures were approved by the Chinese Animal Welfare Act, the Guidance for Animal Experimentation of Capital Medical University (Process No. AEEI‐2018‐200 and AEEI‐2019‐097). Every effort was made to reduce the number of animals used and minimize their suffering.

### 2.2 Epilepsy models and drug administration

The epileptogenesis process of the epilepsy model used in this study can be divided into 3 distinct periods: an acute period that built up progressively into status epilepticus (SE), lasting at least 6‐12 hours; a latent period with a progressive normalization of electroencephalography and behaviour between the acute and chronic period; and a chronic period with spontaneous recurrent seizures about 20‐40 days after SE.^23^ In this study, an epileptic model induced by an intra‐amygdaloid injection of KA was established as described previously.^24^ Briefly, 0.7 μL (1 μg/μL, Sigma‐Aldrich) was injected into the right amygdala (2.76 mm posterior to the bregma, 4.5 mm lateral from the midline and 8.6 mm ventral to the bregma) of the rats, and 0.3 μL KA was injected into the right amygdala (0.94 mm posterior to the bregma, 2.75 mm lateral to the midline and 4.75 mm ventral to the bregma) of the mice. Sham‐operated controls received the same volume of an intra‐amygdala saline injection. Rats at 1, 3, 7 and 10 days after KA injection were used as the latent period, and rats at 30 and 60 days after KA injection were used as the chronic period of epileptogenesis. The STAT3 inhibitor, AG490, was intraperitoneally injected (i.p., 4 mL/kg, Selleck Chemical) 30 minutes before KA injection.

### 2.3 RNA overexpressing and silencing

H19 was overexpressed or silenced using an adeno‐associated viral (AAV) delivery vector constructed by GeneChem Co. as previously described.^21^ A CMV‐bGlobin‐eGFP‐rno was used for AAV‐Let‐7b packaging. The empty AAV vectors encoding EGFP (AAV‐NC) were used as sham controls. The AAV vector was stereotaxically injected as previously described.^22^ A total of 6 μL of the AAV vector were injected into the right dorsal hippocampus (3.12 mm posterior to the bregma, 3.0 mm lateral from the midline and 3.4 mm ventral to the bregma) and ventral hippocampus (5.04 mm posterior to the bregma, 5.0 mm lateral from the midline and 6.4 mm ventral to the bregma) (3 μL at each location in the dorsal‐ventral plane).

### 2.4 Fluorescence in situ hybridization

Paraffin‐embedded sections (4‐μm thick) were deparaffinized, dehydrated and treated with 1 M sodium thiocyanate. The sections were then digested in a pepsin solution, fixed in 4% formaldehyde, dehydrated by sequential immersion in 70%, 85% and 100% ethanol and air‐dried. The sections were incubated with a digoxin (DIG)‐labeled probe (5′‐DIG‐CTCCTGCCAGACTCCAGATGTCCAAGGTGCTCC‐3′ for human H19, 5′‐DIG‐CTTTCCCGTTCTCCGGCCATCGAAGCT‐3′ for rat H19 and 5′‐DIG‐AACCACACAACCTACTACCTCA‐3′ for rat let‐7b) followed by incubation with a DyLight 594‐conjugated IgG fraction (Abcam; ab96873) coupled with a monoclonal mouse anti‐DIG antibody (Abcam; ab116590). The nuclei were counterstained with DAPI. The sections were scanned and analysed as described above.

### 2.5 Statistical analysis

All statistical analyses were performed using GraphPad Prism software (version 7.0; GraphPad Software). A comparison of the data for two different groups was performed using a Student's *t* test or a Wilcoxon Mann‐Whitney test. Statistical comparisons for multiple groups were analysed using a one‐way analysis of variance (ANOVA) and was followed by an appropriate post hoc test. Data are expressed as the mean ± standard error of the mean (SEM). Significance was accepted at *P* < .05.

For a description of the other methods used in this study, see Appendix S1.

## 3 RESULTS

### 3.1 Status epilepticus‐induced hypoxia promoted H19 expression

**FIGURE 1:**
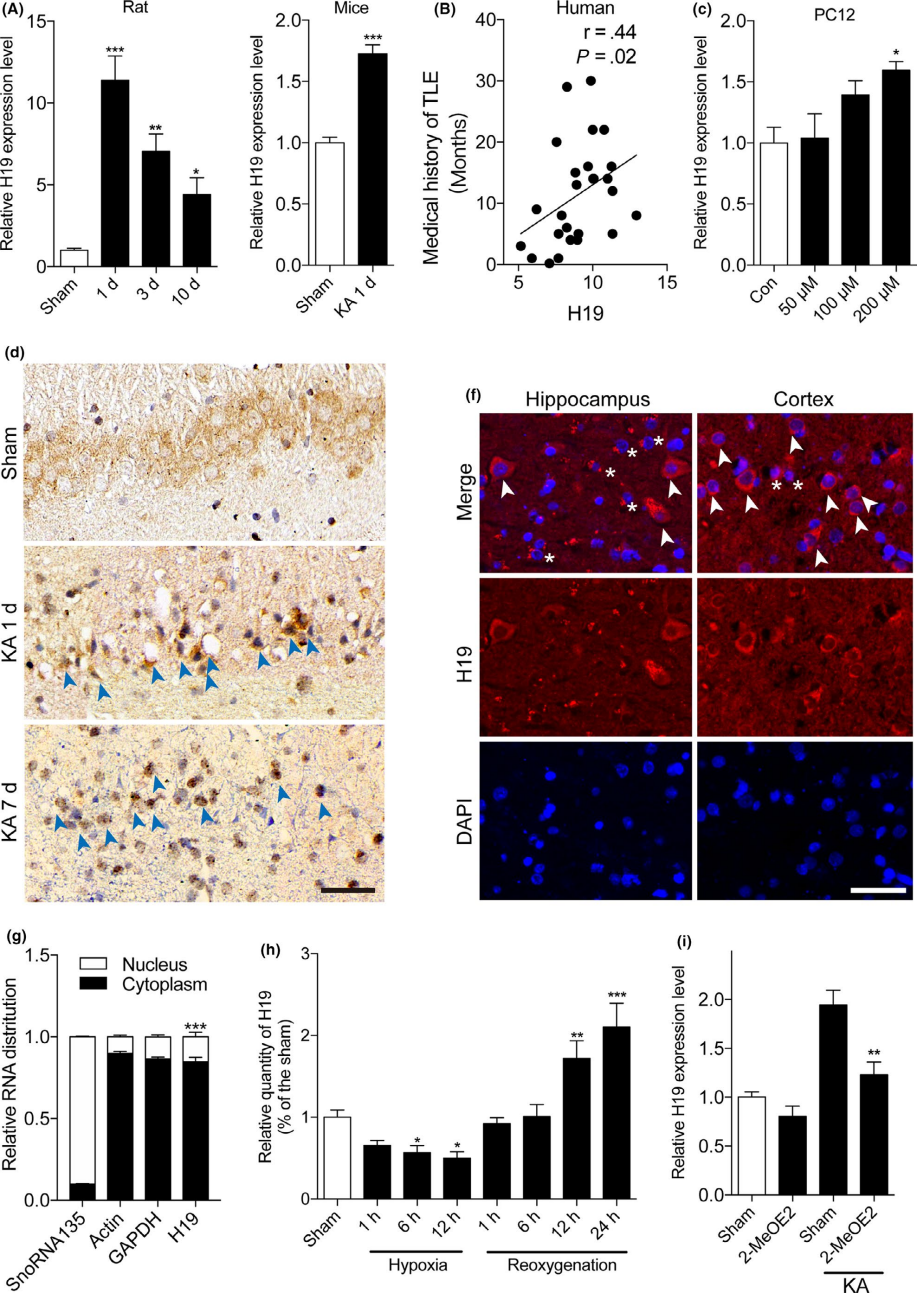
Status epilepticus (SE)‐induced hypoxia promoted H19 expression. The expression of lncRNA H19 was determined by qPCR in the hippocampus from (A) rats at 1, 3 and 10 d post‐SE (n = 5‐7) and (B) mice at 1 d post‐SE (n = 4). C, Correlations according to Pearson's coefficient between H19 expression and the medical history of TLE patients (n = 24). D, H19 expression in PC12 cells treated with different doses of KA as determined by qPCR (n = 3). E, Representative images of the in situ hybridization staining for H19 in the hippocampus of rats at 1 and 7 d post‐SE (scale bar = 50 μm). Arrows indicate H19 positive neurons. F, Fluorescence in situ hybridization assay for H19 in the hippocampus and cortex of TLE patients (scale bar = 50 μm). Arrows and asterisks indicate neurons and astrocytes, respectively. G, Quantification of H19 in both the cytoplasmic and nuclear extract of the hippocampus of the mice. SnoRNA135 was used as the internal nuclear control, and actin and GAPDH were used as the internal cytoplasmic control. H, H19 expression in PC12 cells under hypoxia for 1, 6 and 12 h and reoxygenation for 1, 6, 12 and 24 h, as determined by qPCR (n = 3). I, H19 expression in PC12 cells treated with the HIF‐1α inhibitor, 2‐MeOE2, 30 minutes before KA insult (n = 3). Data represent the mean ± SEM. *P < .05; **P < .01; ***P < .001

Intra‐amygdala injection of KA‐induced epilepsy models in rats and mice was used for the qPCR analyses of H19 expression. H19 expression was significantly increased in the hippocampus of the rats at 1, 3 and 10 days after KA injection (Figure 1A) and epileptic mice on day 1 following KA injection (Figure 1B). These results were consistent with that of our previous report.^21^ The expression of H19 in the hippocampal samples from patients with TLE was examined by qPCR. The correlation analysis showed that H19 was positively correlated with the medical history of TLE patients (Figure 1C). Moreover, the expression of H19 in KA‐treated PC12 cells was also increased (Figure 1D). Further fluorescence in situ hybridization (FISH) experiments on the hippocampus of the rats and TLE patients also confirmed the primary localization of H19 in the neuron cytoplasm and increase of H19 in the hippocampus of rats during epileptogenesis (Figure 1E,F). In addition, both the cytoplasmic and nuclear RNA was isolated from the hippocampus of the mice and the H19 expression in each fraction was detected by qPCR. The results showed that compared with SnoRNA135 (mostly expressed in the nucleus), actin and GAPDH (mostly expressed in the cytoplasm), H19 was mainly expressed in the cytoplasm of the neuron (Figure 1G).

It is well‐established that SE is associated with severe hypoxia. Previous studies have suggested that hypoxia and the transcription factor, hypoxia‐inducible factor‐1α (HIF‐1α), activates the expression of H19.^7^, ^25^ Therefore, we hypothesized that SE‐induced hypoxia and HIF‐1α might activate the expression of H19. To test this presumption, we examined the time course of hypoxia cells in rats surviving to SE, which showed a significant increase of anoxic neurons in the hippocampus at one day after SE (Figure S1). The Western blot and immunofluorescence analyses showed that the level of HIF‐1α protein expression peaked at day 1 and subsequently decreased on days 3, 10 and 30 after SE (Figure S2A–C). The correlation analysis showed that H19 and HIF‐1α had a positive correlation in the rat hippocampus (Figure S2D). Next, the expression patterns of H19 were tested in an in vitro study with cultured PC12 cells under hypoxia and reoxygenation. As shown in Figure 1H, hypoxia suppressed H19 expression, while reoxygenation restored and promoted H19 expression. Inhibiting HIF‐1α activity by 2‐methoxyestradiol (2ME2) in the PC12 cells also inhibited the expression of H19 (Figure 1I). These data suggested that epileptic seizure‐induced hypoxia might lead to H19 expression in the neurons through HIF‐1α.

### 3.2 H19 aggravated epileptic seizures, memory impairment and mossy fibre sprouting in a rat model of TLE

**FIGURE 2:**
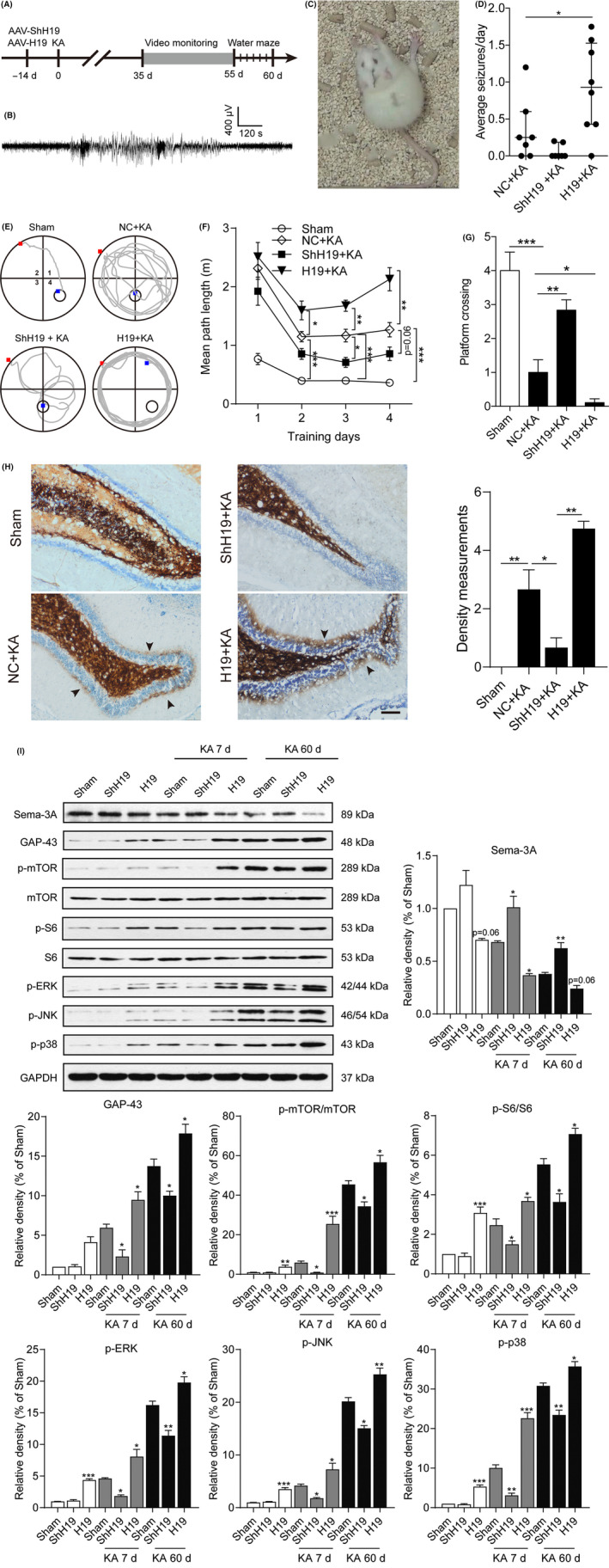
H19 aggravated epileptic seizures, memory impairment and mossy fibre sprouting in a rat model of TLE. A, Experimental timeline of the video monitoring of spontaneous recurrent seizures (SRS) and Morris water maze. B, C, A representative SRS recorded in the EEG (B) and video monitoring (C) of epileptic rats. D, The mean daily frequency of SRS per rat in H19 overexpressed and silenced rats from day 35 to day 55 after SE were recorded by video monitoring (n = 7‐8). E, A representative schematic diagram of H19 overexpressed and silenced rats subjected to the Morris water maze test for a designed time for five consecutive days (n = 11‐12). F, The mean path length and (G) the platform crossing times on the fifth day after the hidden platform was removed. H, Representative Timm and Nissl double staining photomicrographs of the hippocampus from the H19 overexpressed and silenced rats at 60 d after SE (scale bar = 50 µm). The arrows indicate the Timm staining granules in the supragranular region. The right panel shows the Timm score analysis (n = 4‐5). I, Quantification of the protein level of MFS molecular markers, Sema‐3A and GAP‐43, and the mTOR and MAPK signalling molecular markers in the hippocampus of the H19 overexpressed and H19 silenced rats at 7 and 60 d after KA treatment (n = 4‐5). The protein bands were quantified by densitometry and normalized to the level of GAPDH. Data represent the mean ± SEM. *P < .05; **P < .01; ***P < .001

Video camera monitoring and the Morris water maze test were performed to investigate the effects of H19 on spontaneous recurrent seizures, as well as the spatial learning and memory function of the epileptic rats, respectively (Figure 2A). Although knockdown of H19 did not significantly alleviate the spontaneous recurrent seizures (SRS) of epileptic rats, overexpression of H19 increased the SRS of epileptic rats compared to the sham group (Figure 2B‐D). The Morris water maze test showed that the epileptic rats displayed a learning deficiency with prolonged path length compared to the rats treated with empty vectors. Overexpression of H19 increased the path length and the number of times that the epileptic rats crossed the platform. However, knockdown of H19 prevented the learning deficiency with a decreased path length compared to the rats treated with the empty vectors (Figure 2E‐G).

Mossy fibre sprouting (MFS) is one of the most frequently observed hallmarks in patients^26^, ^27^, ^28^ and laboratory animal models with TLE.^29^, ^30^, ^31^ In the present study, the role of H19 on the MFS of TLE rats was also evaluated using Timm staining. In the sham rats, there were no Timm‐stained granules in the supragranular region in the hippocampus. At 60 days post‐SE, epileptic rats treated with empty vectors displayed mossy fibres that filled the hilus, moderate granules in the supragranular regions of epileptic rats (Figure 2H). Knockdown of H19 prevented mossy fibre sprouting compared to the sham rats treated with the empty AAV vectors. In contrast, overexpression of H19 resulted in a much denser and darker stained band in the regions at 60 days post‐SE injection (Figure 2H). GAP‐43 and Sema‐3A are molecular markers of MFS, as GAP‐4 promoted MFS, whereas Sema‐3A inhibited MFS.^32^, ^33^ Quantification of the levels of GAP‐43 and Sema‐3A protein expression after overexpression and knockdown of H19 also confirmed the Timm staining results. The expression of GAP‐43 and Sema‐3A was further influenced by overexpression of H19 at 7 and 60 days after SE, while the knockdown of H19 prevented the SE‐induced changes (Figure 2I). In addition, H19 also promoted mTOR (p‐mTOR, mTOR, p‐S6 and S6) and MAPK (p‐ERK, p‐JNK and p‐P38) signalling (Figure 2I), which were active in the hippocampus of pilocarpine^34^, ^35^ or KA^36^ induced rat models of TLE, as well as in the hippocampus of patients with TLE.^37^, ^38^, ^39^ Together, these results indicated that H19 aggravated epileptogenesis.

### 3.3 H19 binding to let‐7b was decreased in the hippocampus of rats during epileptogenesis

**FIGURE 3:**
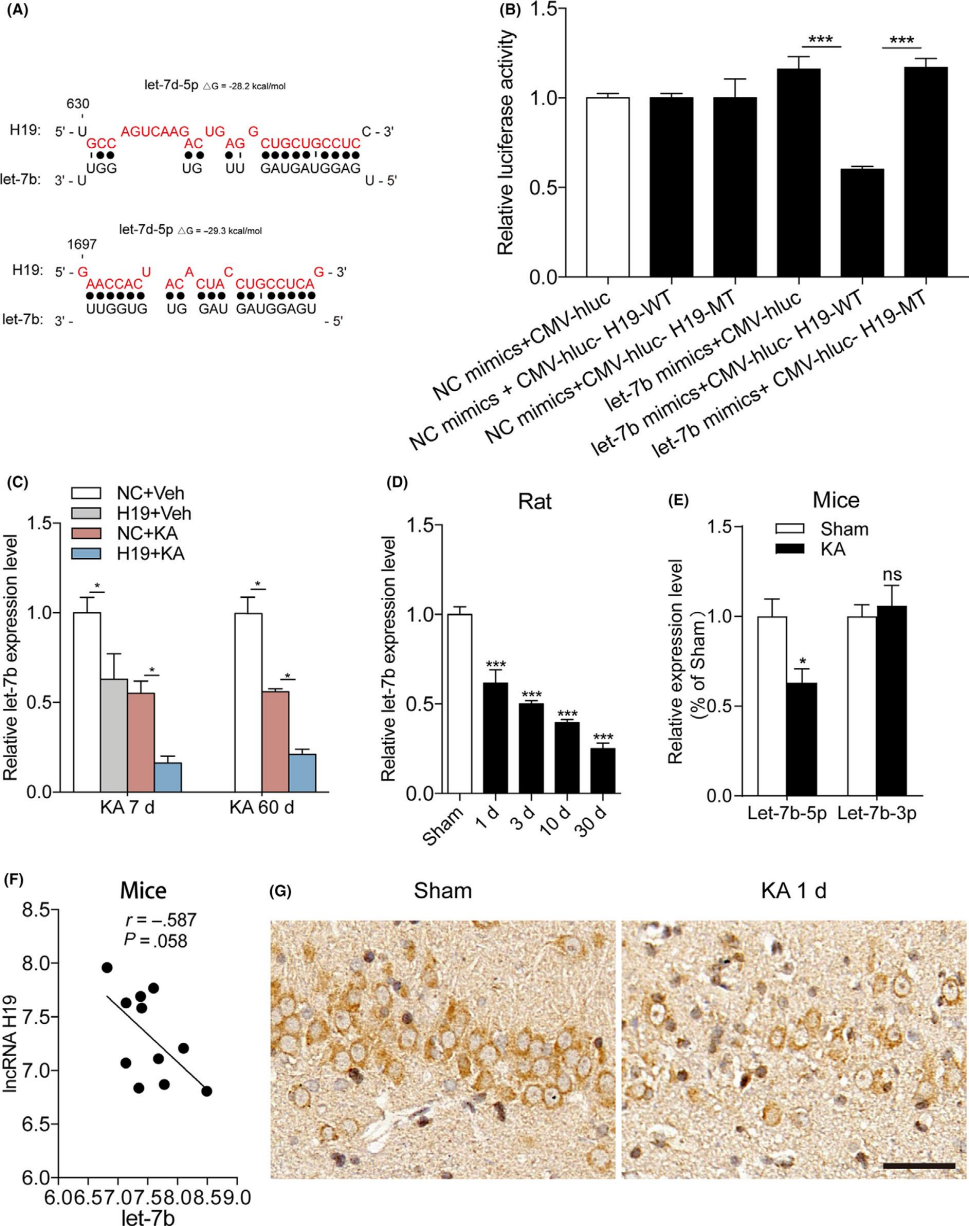
The H19 binding let‐7b was decreased in the hippocampus of rats during epileptogenesis. A, Complementarity between the let‐7b seed sequence and the whole sequence of H19 of rats predicted by RNAhybrid. Mutant H19 (H19‐MT) was generated by mutating the putative let‐7b‐binding site (in red). B, Luciferase reporter gene assay for the interactions between let‐7b and its binding sites or mutated binding sites of H19 in HEK293 cells. The relative luciferase activity was normalized against the firefly luciferase values (n = 6). C, qPCR quantification of let‐7b levels in the hippocampus of rats overexpressing H19 with or without KA treatment for 7 and 60 d (n = 8). D, The expression of let‐7b was determined by qPCR in the hippocampus of rats at 1, 3, 10 and 30 d following the KA injection (n = 5‐6). E, Quantification of let‐7b (5p and 3p) expression in the hippocampus of mice at 1 d after KA injection (n = 5). F, Correlations according to Pearson's coefficient between the level of H19 and let‐7b in the hippocampus from mice under SE at 1 d after surgery (n = 5). G, Representative images of the in situ hybridization staining for let‐7b in the hippocampus of rats at 1 d after SE (scale bar = 50 µm). Data represent the mean ± SEM. *P < .05, ***P < .001

A bioinformatics analysis and luciferase assay demonstrated that H19 harbours binding sites for let‐7b‐5p (called as let‐7b below) (Figure 3A). The luciferase assay demonstrated that the co‐transfection of the let‐7b mimic with the plasmid containing a full sequence of H19 consistently produced lower luciferase activity. In contrast, a mutation of the binding sites abolished the repressive effect of let‐7b (Figure 3B). Overexpression of H19 in the in vivo study further verified a strong negative correlation between the levels of let‐7b and H19 in the rats at 7 and 60 days after SE (Figure 3C). The quantity of the let‐7b expression pattern at different periods of the TLE by qPCR showed that let‐7b was significantly decreased at 1, 3, 7 and 30 days following KA injection (Figure 3D). Moreover, let‐7b (−5p) was also decreased in the KA‐induced mouse model at 1 day after SE (Figure 3E). The correlation analysis also showed that H19 and let‐7b had a negative correlation in the hippocampus of the rats at 1 day after SE (Figure 3F). In addition, FISH staining of the rat hippocampus showed that let‐7b was decreased in the hippocampus of rats at 1 day after KA injection and was primarily localized in the neuron cytoplasm (Figure 3G).

### 3.4 The target genes of let‐7b were increased in the hippocampus of the rats post‐SE

**FIGURE 4:**
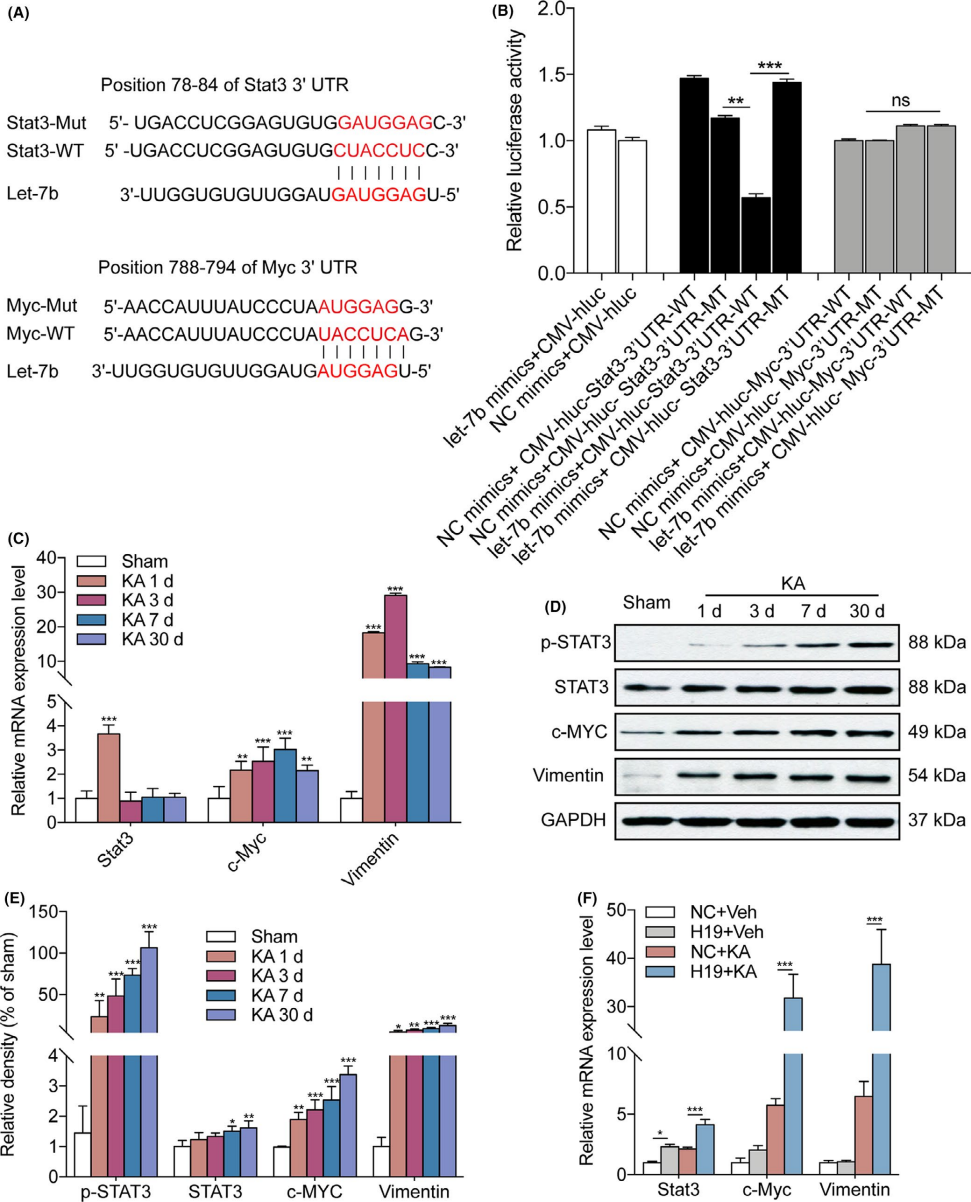
The target genes of let‐7b are increased in the hippocampus of rats post‐SE. A, Complementarity between the let‐7b seed sequence and the 3′‐UTR position of Stat3 and Myc predicted by a computational and bioinformatics‐based approach using TargetScan. Mutant Stat3 (Stat3‐Mut) and mutant Myc (Myc‐Mut) were generated by mutating the putative let‐7b‐binding site (in red). B, A luciferase reporter gene assay for interactions between let‐7b and its binding sites or mutated binding sites of Stat3 and Myc in HEK293 cells. The relative luciferase activity was normalized against the firefly luciferase values (n = 6). C, The level of Stat3, c‐Myc and Vimentin mRNA in the hippocampus of rats at 1, 3, 7 and 30 d after KA injection (n = 5). D, E, The levels of p‐Stat3, Stat3, c‐Myc and Vimentin protein expression in the hippocampus of rats at 1, 3, 7 and 30 d after KA injection (n = 5). Protein bands were normalized to the level of GAPDH. F, qPCR quantification of the level of Stat3, c‐Myc and Vimentin expression in the hippocampus of H19 overexpressed rats with or without KA treatment for 7 d (n = 8). Data represent the mean ± SEM. *P < .05; **P < .01; ***P < .001

**FIGURE 5:**
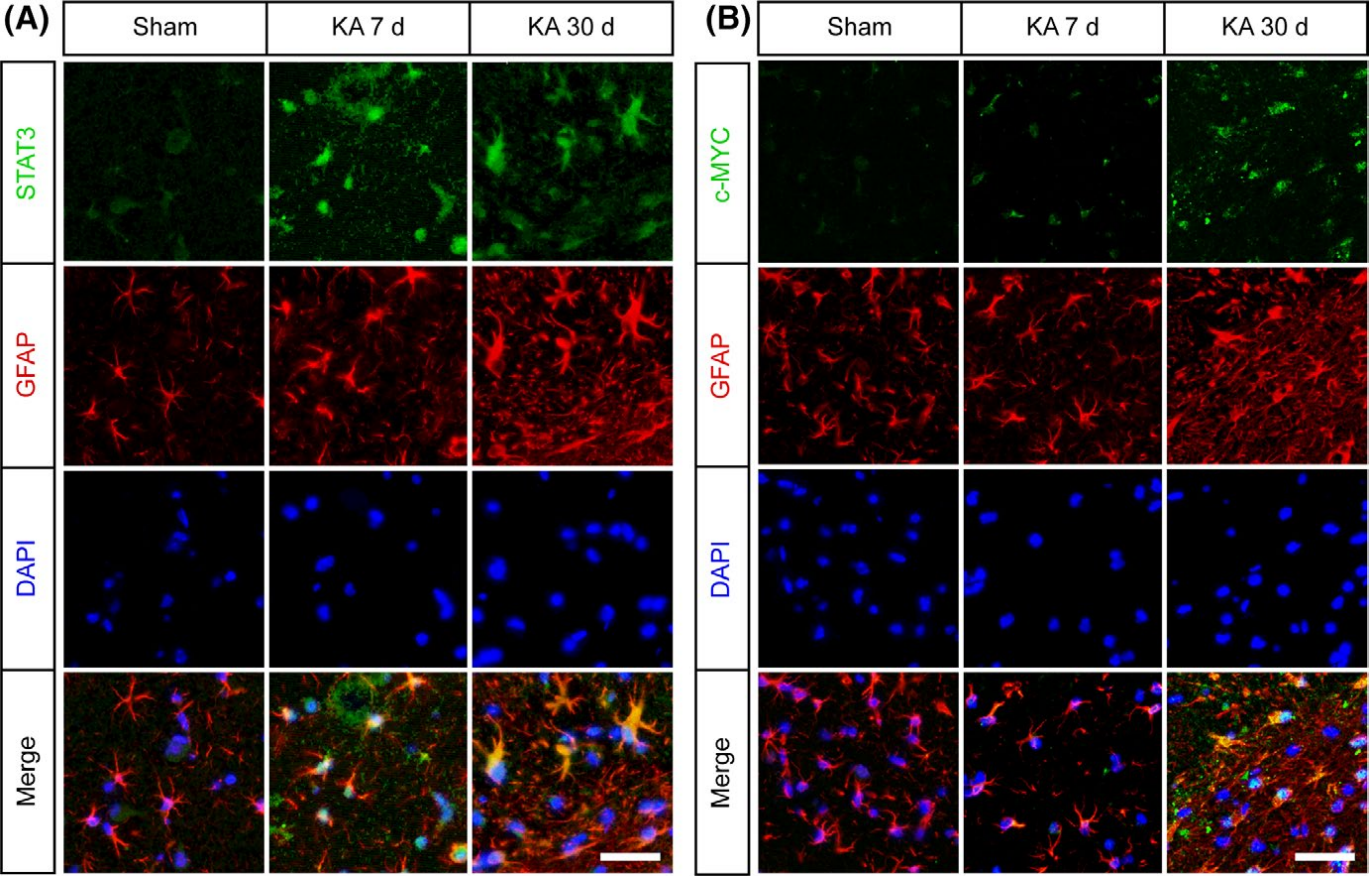
Stat3 and c‐Myc were increased in the astrocytes of the hippocampus of rats during epileptogenesis. Double labelling of (A) Stat3 and (B) c‐Myc with GFAP, respectively, by immunofluorescence in the hippocampus of rats at 7 and 30 d post‐SE (n = 3) (scale bars = 50 μm)

Previous studies indicated that Stat3 plays a key role in astrocyte proliferation after central nervous system injury.^40^, ^41^ MicroRNA target gene prediction showed that Stat3 and its downstream molecules, c‐Myc, are potential downstream target genes of let‐7b. The luciferase assay confirmed the direct binding of let‐7b to Stat3, but not c‐Myc (Figure 4A,B). The qPCR analysis showed that the level of Stat3, c‐Myc and Vimentin (the marker of astrocyte proliferation) mRNA expression was significantly increased after SE (Figure 4C). Next, Western blotting was performed to detect Stat3, phosphorylated Stat3 (p‐Stat3), c‐Myc and Vimentin at 1, 3, 7 and 30 days after SE. The levels of p‐Stat3, total Stat3 and c‐Myc protein expression were significantly increased in the hippocampus of the rats at 1, 3, 7 and 30 days after KA injection, compared with the sham group (Figure 4D,E). Overexpression of H19 in the in vivo study further verified the strong positive correlation of H19 and Stat3 in the rats at 7 days after SE, although H19 only promoted the expression of c‐Myc and Vimentin under KA treatment (Figure 4F). The immunofluorescence analysis demonstrated that Stat3 (Figure 5A) and c‐Myc (Figure 5B) were increased in the active astrocytes in the hippocampus of rats at 7 and 30 days after SE. These findings suggested that let‐7b was decreased while its target genes were increased in epilepsy.

### 3.5 Let‐7b inhibited glial cell activation by targeting Stat3, and H19 blocked the inhibitory effect of let‐7b in the hippocampus of rats during epileptogenesis

**FIGURE 6:**
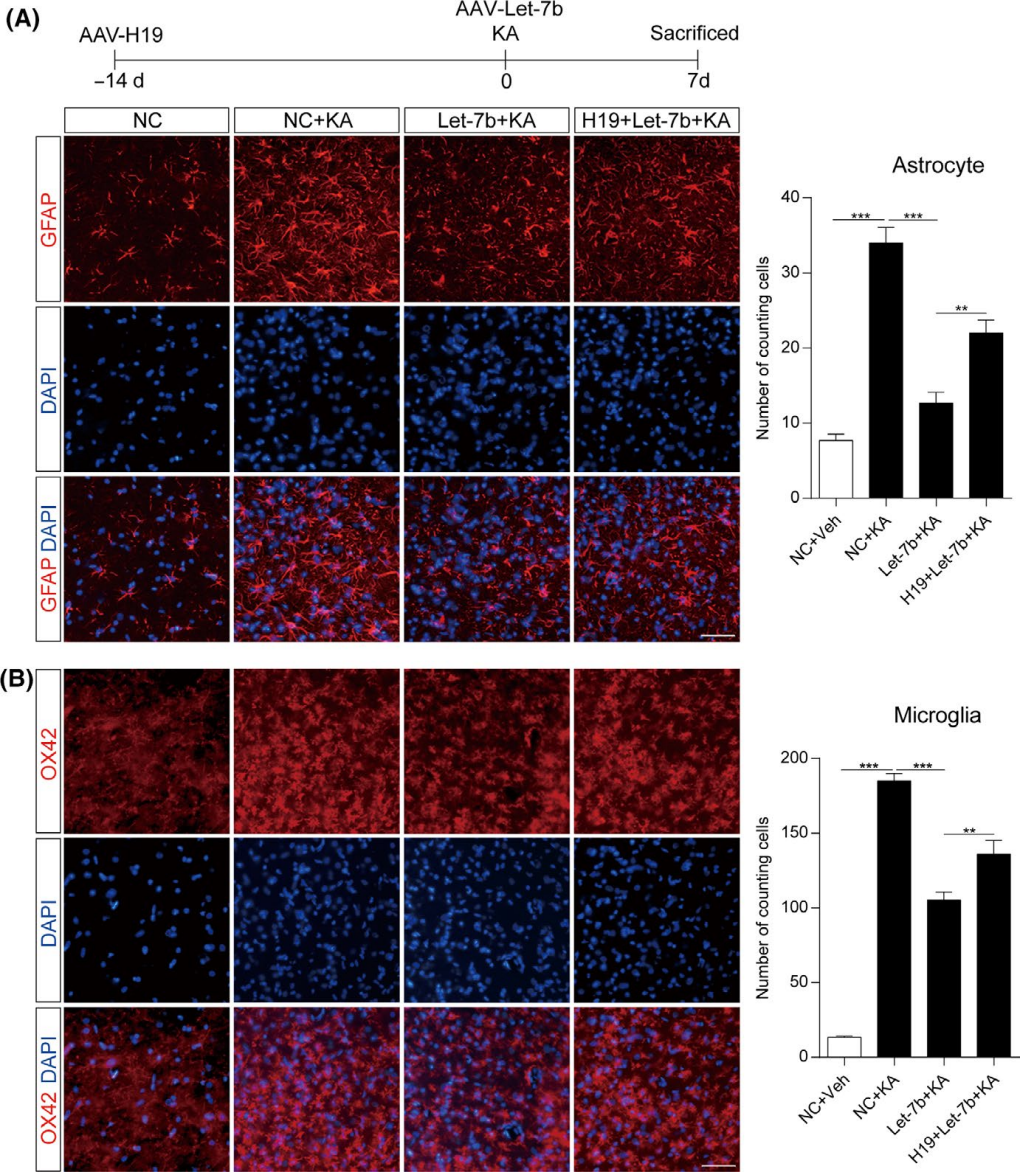
H19 promoted glial cell activation by let‐7b in the hippocampus of rats during epileptogenesis. Representative fluorescence micrographs of (A) GFAP and (B) OX42 expression in the hippocampus of let‐7b and H19 overexpressed rats with or without KA treatment for 7 d (scale bar = 50 μm). The right panels show the cell counts in the hippocampus (n = 5 −6). Data represent the mean ± SEM. **P < .01; ***P < .001

Next, the biological functions of let‐7b were investigated in vivo by overexpressing let‐7b using an AAV delivery system. Astrocyte and microglia activation was evaluated by GFAP and OX42 immunofluorescence and Western blotting. As shown in Figure 6A,B, marked astrocyte and microglia activation was observed in the hippocampus of rats at 7 days after SE compared to the sham control. Moreover, the SE‐induced astrocyte (Figure 6A) and microglia (Figure 6B) activation was partially inhibited by overexpression of let‐7b at 7 days after SE. The inhibitory effect of let‐7b on astrocyte and microglia activation could be reversed by H19 in the hippocampus of rats at 7 days after SE (Figure 6A,B).

**FIGURE 7:**
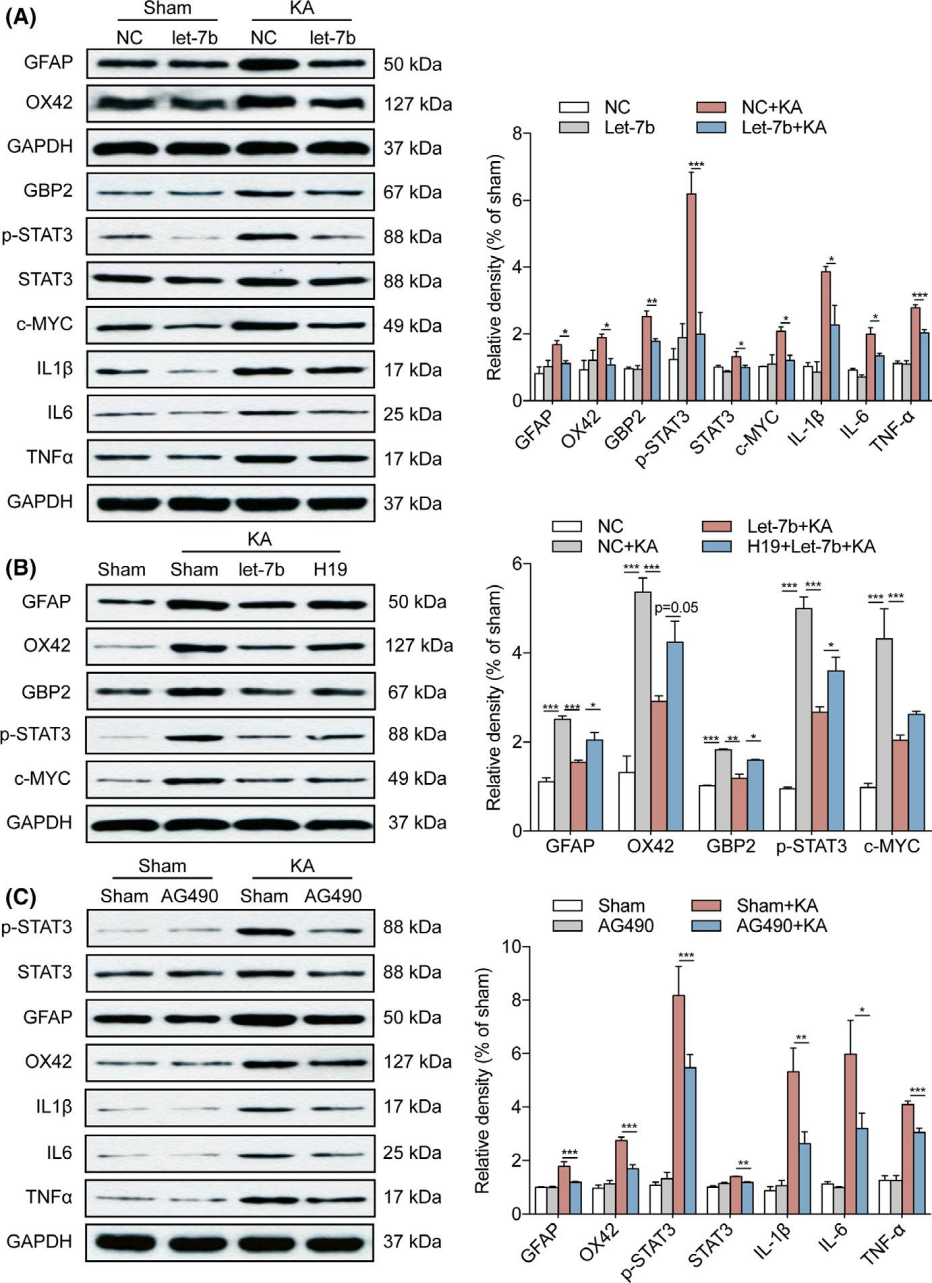
Let‐7b inhibited glial cell activation and inflammatory response by targeting Stat3 and H19 blocked the inhibitive effect of let‐7b on glial cell activation in the hippocampus of rats during epileptogenesis. A, The protein levels of GFAP, OX42, Gbp2, p‐Stat3, Stat3, c‐Myc, IL‐1β, IL‐6 and TNF‐α in the hippocampus of let‐7b overexpressed rats at 7 d post‐SE, as determined by Western blot (n = 4). B, The protein levels of GFAP, OX42, Gbp2, p‐Stat3, Stat3 and c‐Myc in the hippocampus of let‐7b and H19 overexpressed rats with or without KA treatment for 7 days, as determined by Western blot (n = 4). C, Western blot analysis of GFAP, OX42, Gbp2, p‐Stat3, IL‐6 and TNF‐α in the hippocampus of rats treated with AG490 at 7 d post‐SE (n = 4). Protein bands were normalized to the level of GAPDH. Data represent mean ± SEM. *P < .05, **P < .01, ***P < .001

The quantitative analysis of the level of GFAP and OX42 protein expression in the hippocampus further confirmed these results (Figure 7A,B). It has been reported that there are two different subtypes of reactive astrocytes, termed A1 and A2, respectively. A1 astrocytes, which highly upregulate many classical complement cascade genes, are harmful, whereas A2 is protective.^42^, ^43^ Western blotting showed that A1 type maker, Gbp2, was inhibited by let‐7b in the hippocampus of rats at 7 days after SE (Figure 7A) and H19 could reverse its expression (Figure 7B). Since activated glia can release proinflammatory cytokines, we also examined the proinflammatory cytokines in the hippocampus. As shown in Figure 7A, compared to the sham group, KA‐induced SE stimulated the release of IL‐1β, IL‐6 and TNF‐α, whereas overexpressing let‐7b prevented the SE‐induced increase in the elevation of IL‐1β, IL‐6 and TNF‐α levels in the hippocampus of rats at 7 days post‐SE (Figure 7A). Together, these findings showed that lncRNA H19 contributed to hippocampal glial cell activation by sponging let‐7b to target Stat3 during the epileptogenesis of TLE.

The Western blot analysis showed that the expression of Stat3, p‐Stat3 and its downstream effector, c‐Myc, were decreased following overexpressing let‐7b (Figure 7A) and H19 could reverse their expression (Figure 7B). We further explored the role of Stat3 in glial cell activation in epilepsy using the Stat3 inhibitor, AG490. As shown in Figure 7C, KA‐induced SE increased the level of p‐Stat3, GFAP and OX42 protein expression and stimulated the release of IL‐1β, IL‐6 and TNF‐α in the hippocampus at 7 days after SE. AG490, intraperitoneally injected 30 minutes before KA injection, inhibited glial cell activation and the inflammatory response (Figure 7C). These results indicated that let‐7b inhibits the activation of astrocytes and microglia by targeting Stat3 in the hippocampus of the rats during epileptogenesis.

### 3.6 Overexpression of let‐7b alleviated the seizure frequency and severity in the rat model of temporal lobe epilepsy

**FIGURE 8:**
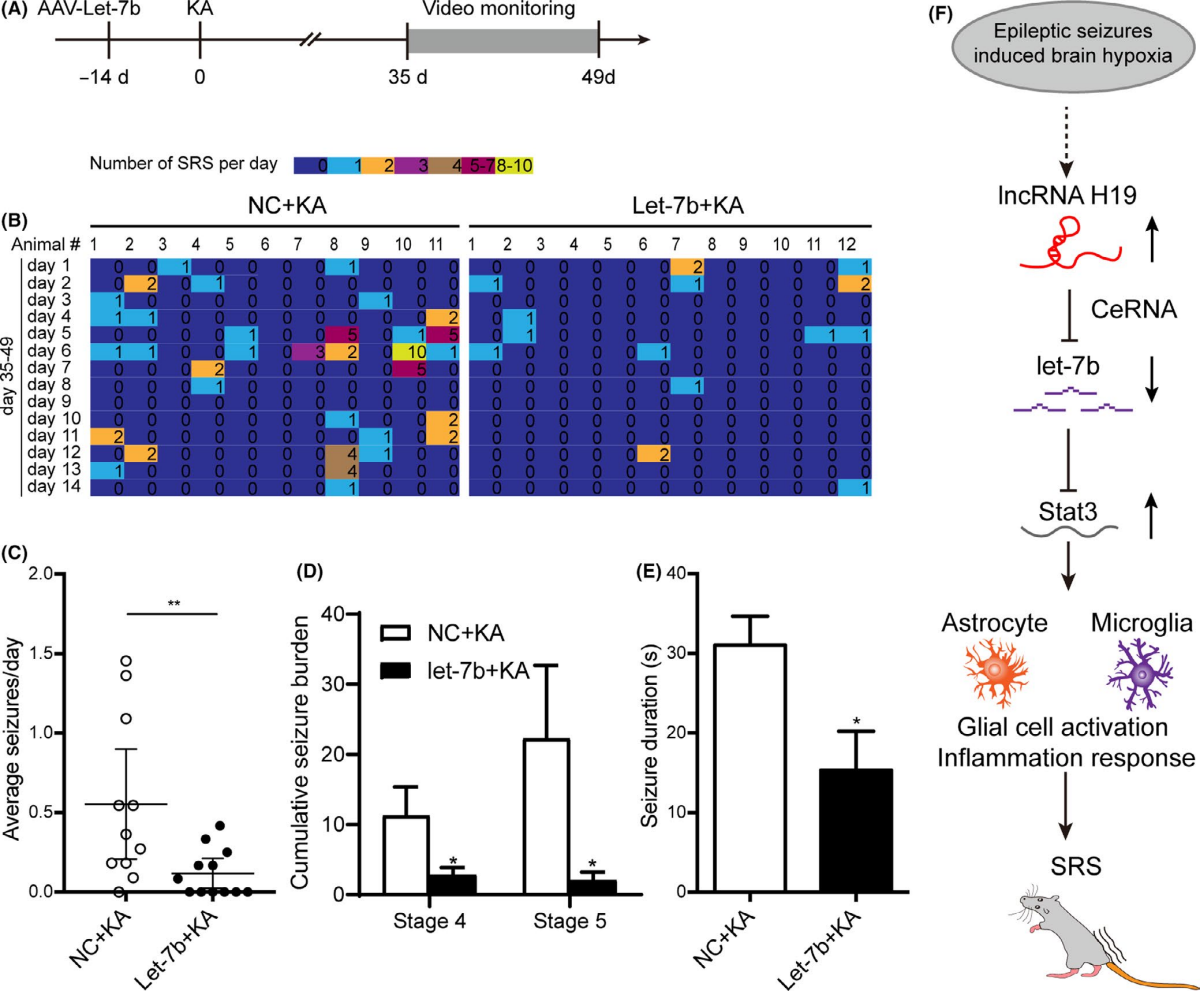
Let‐7b alleviated the frequency and severity of epileptic seizures in a KA‐induced rat model. A, Experimental timeline. B, The SRS number of the epileptic rats with or without overexpression of let‐7b from day 35 to day 49 post‐SE by video monitoring (n = 11‐12). C, The mean daily frequency, (D) average seizure burden and (E) duration of SRS per rat in the epileptic rats with or without overexpression of let‐7b (n = 11‐12). F, SE‐induced brain hypoxia promoted H19 expression. Increased H19 promoted hippocampal glial cell activation by sponging let‐7b to target Stat3 in a rat model of temporal lobe epilepsy. Data represent the mean ± SEM. *P < .05; **P < .01

To investigate whether let‐7b affected SRS during the chronic period, a video monitoring procedure was used to determine the frequency and severity of the chronic epileptic seizures from day 35 to day 49 after KA‐induced SE in rats with or without overexpression of let‐7b (Figure 8A). Strikingly, the let‐7b overexpressed rats displayed a significant decrease in the frequency (Figure 8B,C), average seizure burden (Figure 8D) and seizure duration (Figure 8E) of recurrent epileptic seizures compared with the sham rats. These findings indicated that the let‐7b treatment attenuated SRS severity.

## 4 DISCUSSION

In this study, we found that brain hypoxia‐induced H19 post‐SE contributed to epileptogenesis by aggravating the epileptic seizures, memory impairment and mossy fibre sprouting of the rat model of TLE. H19 could competitively bind let‐7b, which was decreased during the latent period of epileptogenesis. Specifically, overexpression of let‐7b inhibited hippocampal astrocytes and microglia cell activation, inflammatory response and epileptic seizures by targeting Stat3. Moreover, overexpression of H19 reversed the inhibitory effect of let‐7b on glial cell activation. Together, lncRNA H19 could competitively bind let‐7b to promote hippocampal glial cell activation and epileptic seizures by targeting Stat3 in a rat model of temporal lobe epilepsy (Figure 8F). Our study reveals a novel non‐coding RNA‐mediated mechanism in seizure‐induced glial cell activation.

The latent period of epileptogenesis is a slowly developing seizure‐free process, during which pathological molecular and cellular changes happened, finally result in the occurrence of SRS.^44^ In our previous study, we found that H19 was significantly increased during the latent period of epileptogenesis.^21^ Here, the increase in H19 as shown by qPCR and FISH further confirmed our previous results. Furthermore, we also found that H19 was positively related to the clinical medical history of the TLE patients, which demonstrated the close relationship between H19 and TLE. It has been well‐established that epileptic seizures, including SE and spontaneous recurrent seizures, result in a severe hypoxic event in the brain, which could last for at least 1 hour after seizure termination.^2^, ^45^ Moreover, it has been reported that hypoxia is an important factor for the regulation of H19. Studies from Matouk's group have identified hypoxia as a principal inducer of H19 in tumour cells, since overexpression of HIF‐1α enhances H19 expression whereas it is abolished by knockdown of HIF‐1α.^8^ The present study showed that hippocampal neuron hypoxia, as well as the level of HIF‐1α protein expression, peaked at 24 hours after SE, and H19 also peaked at the same time. This indicated that H19 expression was associated with the epileptic seizure‐induced brain hypoxia. The H19 expression pattern in cultured PC12 cells under hypoxia in vitro also suggested this association and HIF‐1α inhibition also blocked H19 expression in vitro. Together, these results demonstrated that epileptic seizure‐induced brain hypoxia might activate H19 expression.

We previously found that H19 aggravates SE‐induced neuron injury and hippocampal glial cell activation in the hippocampus.^20^, ^21^ In the present study, we further confirmed that H19 aggravated epileptic seizures, memory impairment and mossy fibre sprouting in the epileptic rats. We also found that H19 has an impact on several TLE‐associated signalling molecules (eg, MAPK and mTOR), which indicates the broad role of H19 in epileptogenesis.^22^ Taken together, our results showed that H19 was one lncRNA that contributed to epileptogenesis and H19 is implicated in a broad spectrum of epileptogenic processes.

LncRNAs have different mechanisms of action, depending on their subcellular location. LncRNAs located in nuclei usually interact with proteins (eg, transcriptional factors and histones) and DNA sequences, whereas the cytoplasm‐situated lncRNAs mainly function as competing endogenous RNA (ceRNA).^46^ The mechanism of CeRNA, in which mRNA or lncRNA regulates the distribution of miRNA molecules on their targets, thereby imposes an additional level of post‐transcriptional regulation, which is an important modulatory mechanism of lncRNA.^47^, ^48^ In this study, FISH, as well as the cytoplasmic and nuclear RNA isolation analyses, showed that H19 and let7b were both primarily located in the cytoplasm of hippocampal neurons, which provided the fundamental basis of the ceRNA mechanism of H19. Moreover, it was reported that lncRNA H19 can function as a ceRNA to bind a series of microRNAs, including let‐7, to suppress their targets.^11^, ^12^, ^13^ Using a bioinformatics analysis and luciferase assay, Kallen et al demonstrated that H19 harbours binding sites for the microRNA let‐7 family and can bind directly to let‐7 to modulate its availability.^12^ In both this study and our previous work,^21^ we found that H19 had a strong negative correlation with let‐7b, and H19 could directly bind let‐7b in the hippocampus of rats during epileptogenesis.^21^ Therefore, H19 promoted hippocampal glial cell activation as the ceRNA mechanism which bound let‐7b during epileptogenesis.

In the present study, we explored the let‐7b expression pattern in the hippocampus of rats during epileptogenesis to show a significant decrease. Risbud et al explored the microRNA expression profile in the rat hippocampus of pilocarpine‐induced SE using a microRNA array. In that study, the let‐7 family members, including let‐7b, were decreased by more than 50% two days after SE.^49^ In addition, the study by Song et al analysed the expression of let‐7e, another member of the let‐7 family, at different times after SE. They also found that let‐7e was downregulated at 2 and 7 days after SE.^50^ These results were consistent with our findings. There have also been studies that explore the microRNA expression profile in the hippocampus of patients with TLE; however, the expression of let‐7 family members in these studies is inconsistent, as they are increased in some studies^51^ but decreased in others.^52^ Studies on the function of let‐7b have been primarily focused on carcinogenesis, which has been shown to suppress cell proliferation, migration and invasion in several types of cancers, including glioblastoma.^53^, ^54^ In this study, we found that let‐7b displayed an inhibitory effect on glial cell proliferation. The activation of astrocytes and microglia was manifested by changes in the expression, morphology and proliferation of specific markers.^55^, ^56^ In our study, we analysed the level of GFAP and OX42 protein expression along with morphological changes and cellular proliferation. We found that the high expression of glial cell marker protein (GFAP and OX42) and cellular hypertrophy with long, thick processes, which was consistent with previous reports.^55^, ^57^ Despite their predominantly intracellular localization, miRNAs could also go into extracellular compartments (eg, by exosomes).^58^, ^59^ Thus, it is not surprising that the neuron‐derived miRNAs have an effect on the glial cells.

Stat3, a member of the JAK/STAT signalling pathway, plays a key role in astrocyte proliferation following central nervous system injury^40^, ^41^ and SE.^57^ In our previous study, we found that Stat3 upregulation and its downstream effector, c‐Myc, contributed to glial cell proliferation in the hippocampus of rats during epileptogenesis.^20^ In this study, TargetScan prediction and dual‐luciferase reporter gene assays confirmed that Stat3 was one of the target genes of let‐7b, which is consistent with previous reports.^60^ We also found that Stat3 and c‐Myc were markedly increased in the hippocampal astrocytes of rats during epileptogenesis. In addition, overexpression of let‐7b inhibited the total Stat3, as well as the level of phosphorylated Stat3. Together, these results indicated that the decrease of let‐7b in the hippocampal neurons and subsequent loss of its inhibition on Stat3 in the astrocytes in the latent period of TLE results in the glia cell reactivation of rats during epileptogenesis.

However, the limitation of this study was that we did not use continuous video monitoring combined with EEG recording to evaluate the effects of H19 and let‐7b, which weakened our conclusion. Meanwhile, silencing of H19, as well as upregulation of let‐7b, was only performed prior to SE induction thereby only showing a neuroprotective effect but not an antiepileptogenic effect. Further studies to modulate H19 or let‐7b after the initial precipitating injury or during SRS period are needed to translate the results into clinic treatment. In summary, our findings suggested that the H19/let‐7b/Stat3 axis contributes to hippocampal glial cell activation in the epileptogenesis of TLE and provides a new target in developing non‐coding RNA‐based strategies to reduce seizure‐induced brain injury.

## CONFLICT OF INTEREST

The authors declare no competing financial interest.

## AUTHOR CONTRIBUTIONS

Chun‐Lei Han and Yun‐Peng Liu performed the experiments and drafted the manuscript. Chen‐Jia Guo established the epilepsy model. Kai‐Liang Wang performed the intro‐hippocampus AAV vector injection. Ting‐Ting Du performed immunofluorescence image analysis. Ying Jiang performed cell experiments. Xiao‐Qiu Shao performed the electroencephalographic recording, designed the study and revised the manuscript. Jian‐Guo Zhang and Fan‐Gang Meng designed the experiments. All authors have read and approved the final version of the manuscript.

## Supporting information

Appendix S1

## Data Availability

The data that support the findings of this study are available from the corresponding author upon reasonable request.
