# Supplementary material for: The lncRNA H19 binding to let‐7b promotes hippocampal glial cell activation and epileptic seizures by targeting Stat3 in a rat model of temporal lobe epilepsy
Source: Cell Prolif. 2020 Jul 10;53(8):e12856. doi: 10.1111/cpr.12856 (PMC7445408; doi:10.1111/cpr.12856)
Supplement: Supplementary file 1 — Appendix S1 [file CPR-53-e12856-s001.docx]

**Appendix S1**

**Figure S1**


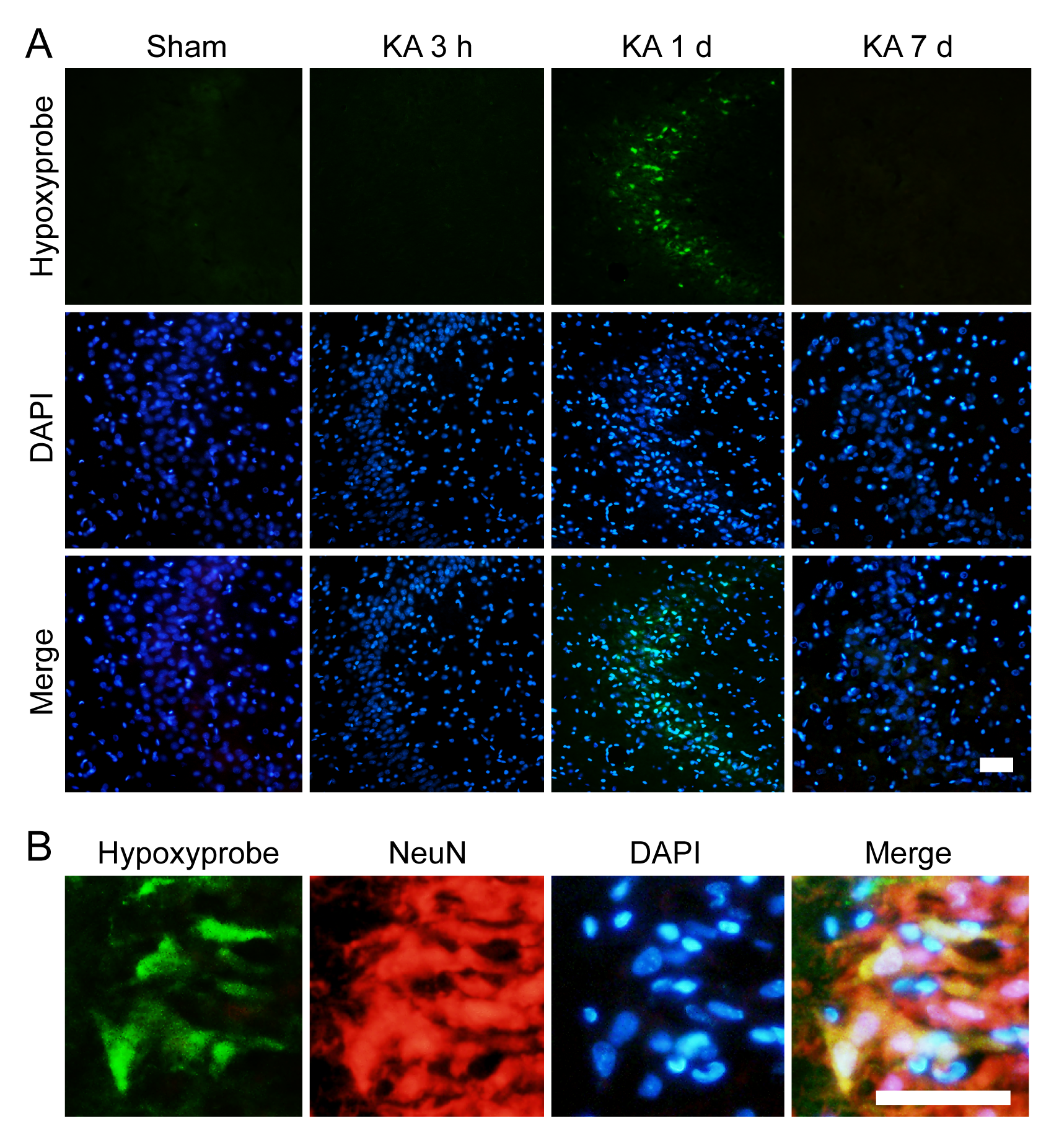


**Figure S1. The hypoxia neuron in the hippocampus peaked at 1 day after KA-induced status epilepticus (SE).** (A) The photomicrographs illustrating hypoxia neurons in the hippocampus of KA-treated rats at 3 h, 1 d, and 3 d post-SE by hypoxyprobe staining. (B) Double-labeling of hypoxyprobe with NeuN by immunofluorescence in the hippocampus of rats at 1 d post-SE (n = 3). Scale bar = 50 μm.

**Figure S2**


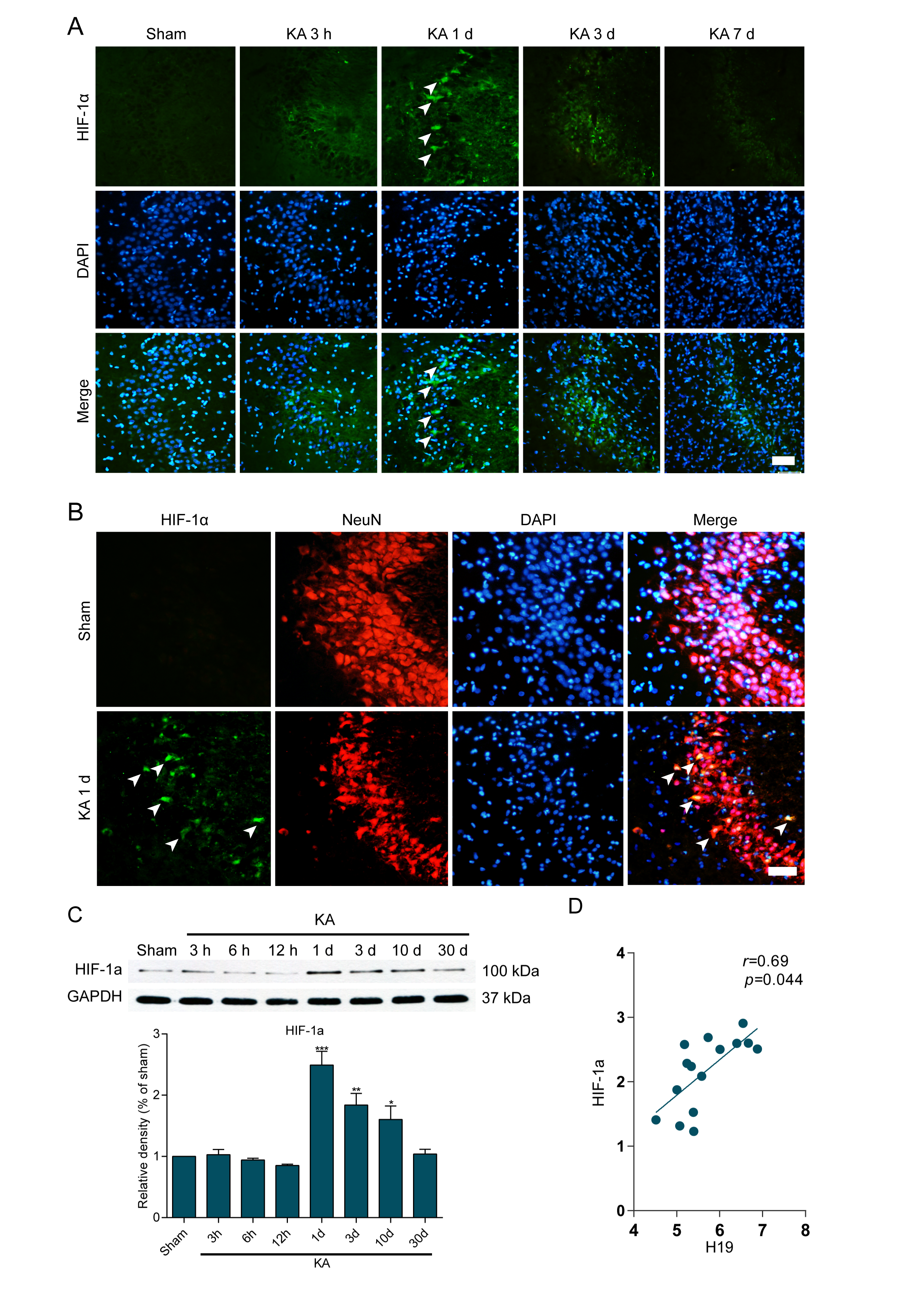


**Figure S2. The expression of HIF-1α was increased in the hippocampal neurons of rats during epileptogenesis.** (A) Representative fluorescence micrographs of HIF-1α expression in the CA3 subfield of the hippocampus of rats at 3 h, 1 d, 3 d, and 7 d post-SE (Scale bar = 50 μm). (B) Double-labeling of HIF-a with NeuN by immunofluorescence in the hippocampus from KA-induced epileptic rats at 1 d post-SE (n=3) (Scale bars=50 μm). Arrows indicate the HIF-1α positive neurons. (C) The protein levels of HIF-1α in the hippocampus of rats at 3 h, 6 h, 12 h, and 1 d, 3 d, 10 d, and 30 d after KA injection, as determined by western blot (n = 4). (D) Correlations according to Pearson coefficient between H19 and HIF-1α in the hippocampus of rats (n=15). Data represent mean ± SEM. *P < 0.05, **P < 0.01, ***P < 0.001.

**Supplementary materials and methods**

**1. Human samples and ethics statement**

The surgically resected hippocampi used in this study were obtained from patients with intractable TLE who underwent surgical treatment at Beijing Tiantan Hospital. All patients had a diagnosis of pharmacoresistant TLE with hippocampal sclerosis. Control hippocampal tissues were obtained from an autopsy of patients without a history of epilepsy or other neurological diseases. All autopsies were performed within 8 h following death. All experimental protocols in this study were approved by the Ethics Committee on Human Research at Capital Medical University. Written informed consent was obtained from each patient for the use of brain tissues for research purposes.

**2. Cell culture and treatments**

Rat adrenal pheochromocytoma cells (PC12, well-differentiated) were cultured at 37 °C and 5% CO2 in 1640 medium containing 10% fetal bovine serum (both from Gibco, Grand Island, NY, USA). The cells were passaged every three days at a density of 1×10^6^ cells/ml. Hypoxia was induced by culture of cells at 37°C in an incubator (Pro-Ox, Biospherix) with 1% O_2_ and 5% CO_2_ balanced with N_2_. HIF-1α inhibitor, 2-MeOE2 (0.5 μM, Selleck Chemicals, Texas, USA), was administrated 30 min before KA (100 μM, Sigma–Aldrich, St. Louis, MO, USA) insult. The cells were then harvested 24 hours after KA insult for qPCR analysis.

**2. Quantitative real-time PCR**

Routine procedures were carried out as previously described ^1^. The primer sequences were 5'-GATGGAGAGGACAGAAGGACAGT-3', and 5'-GAGAGCAGCAGAGATGTGTTAGC-3' for rat H19; 5'- CCTCAAGATGAAAGAAATGGTGCTA-3' and 5'- TCAGAACGAGACGGACTTAAAGAA-3' for mouse H19; 5'- TGCTGCACTTTACAACCACTG-3' and 5'-ATGGTGTCTTTGATGTTGGGC-3' for Human H19; 5′- CAAAGAAAACATGGCCGGCA -3' and 5'- GGGGGCTTTGTGCTTAGGAT-3' for Stat3; 5'-GCTCTCCGTCCTATGTTGCG-3' and 5'- TCGGAGACCAGTTTGGCAG -3' for c-Myc; 5'- AATTGCAGGAGCTGAATGAC-3' and 5'-AATGACTGCAGGGTGCTCTC-3' for Vimentin; 5'- ACTGGAAAGCCGAAACTCTTCATCA -3', and 5'- GGAAGTCGGCCTCCACTGGTATC -3' for Casp3; 5'- TTAGCGAGTGGGTCACAGCGG -3' and 5'- CGAGTTCCAGTGCCTTTTGTCTATG -3' for Bdnf; 5'- GTCGGTCGGTGCCATCATT -3' and 5- TGAGCCTTGAACTGAGCCTCTG -3' for Igfbp3; 5’- GCTGGTGCCGAGTATGTT -3’ and 5’- CAGAAGGTGCGGAGATGA -3' for rat GAPDH; 5'- CTGGGCTACACTGAGCACC -3' and 5'- AAGTGGTCGTTGAGGGCAATG -3' for human GAPDH. Each sample was determined in triplicate. The PCR products were confirmed by a melting curve analysis. The relative expression was normalized to GAPDH or U6 using the 2^−ΔΔCt^ method.

**3. Western blot analysis**

Routine procedures were carried out as previously described ^2^. The following primary antibodies were used for the western blot: mouse monoclonal anti-HIF-1α (1:1000, ab1); rabbit monoclonal anti-p-Stat3 (1:500, ab76315); rabbit monoclonal anti-Stat3 (1:500, ab68153); rabbit polyclonal anti-c-Myc (1:500, ab39688); rabbit polyclonal anti-Vimentin (1:1000, ab92547); rabbit polyclonal anti-GFAP (1:1000, ab7260); mouse monoclonal anti-OX42 (1:50, ab1211); rabbit polyclonal anti-interleukin (IL)-1β (1:500, ab9722); mouse monoclonal anti-IL-6 (1:500, ab9324); and rabbit polyclonal anti-tumor necrosis factor (TNF)-α (1:500, ab6671) (all from Abcam, Cambridge, MA, USA). The rabbit monoclonal anti-glyceraldehyde 3-phosphate dehydrogenase (GAPDH) antibody (1:3,000, ab181602, Abcam) was used as a control. Quantification of the blots was performed using an Epson V330 Photo scanner (Seiko Epson Co., Nagano, Japan) and analyzed with Quantity One software (Bio-Rad, Hercules, CA, USA).

**4.** **Immunofluorescence analysis**

Formalin-fixed, paraffin-embedded tissue sections (4-μm thick) were dried, washed, permeabilized, blocked in 5% goat serum, and incubated overnight with antibodies against HIF-1α (1:150, ab1), NeuN (1:500; Abcam/ab177487), Stat3 (1:140, ab68153), c-Myc (1:50, ab39688), OX42 (ab1211, 1:200), and glial fibrillary acidic protein (GFAP) (ab7260, 1:200) (all from Abcam, Cambridge, MA, USA). The immunolabeled sections were washed and incubated with goat secondary antibodies conjugated to either Alexa Fluor 594 or Alexa Fluor 488 (Merck Biosciences, Nottingham, UK). Sections were mounted with medium containing 4',6-diamidino-2-phenylindole (DAPI) (Vector Laboratories, Burlingame, CA, USA). The sections were scanned and digitized using Pannoramic MIDI (3D HISTECH, Budapest, Hungary) and the images were analyzed using Pannoramic Viewer software (3D HISTECH).

The hippocampal cellular hypoxia was detected by a HypoxyprobeTM-1 Kit (CHEMICON International, Inc., CA) according to the manufacturer’s recommendation. Briefly, a dose of 60 mg/kg hypoxyprobe-1 was injected (i.p.). Sixty minutes after injection, brain tissue was obtained. For staining, frozen sections were dried, washed, permeabilized, and blocked in 5% goat serum and then incubated overnight with Hypoxyprobe1Mab1 (1:50, CHEMICON International, Inc., CA) or combined with NeuN (1:500; Abcam/ab177487). The sections are then incubated for 90 min with Cy-3-conjugated goat anti-mouse antibody 1:150 (Jackson Immuno Research Laboratories) or combine with the secondary goat antibodies conjugated with Alexa Fluor 594 (for NeuN) (BioSciences Ltd).

**5. Timm staining**

Each of rat was transcardially perfused with 150 mL 0.9% sodium chloride, 100 mL 1% sodium sulfide perfusion medium, 100 mL of 4% paraformaldehyde (PFA), and 50 mL 1% sodium sulfide perfusion medium. The sections were developed in the dark for 40−60 min in a solution containing 30 mL of 50% arabic gum, 5 mL of citric acid (51 g/100 mL H_2_O), 5 mL sodium citrate (47 g/100 mL H_2_O), 0.42 g hydroquinone in 7.5 mL H_2_O, and 0.055 g AgNO3 in 7.5 mL H_2_O. After Timm staining, the slides were double stained using Nissl staining. The slides were then washed, dehydrated in alcohol, cleared in xylene, and mounted on slides with neutral balsam. The extent of mossy fiber sprouting was evaluated by one individual according to a standardized 0−5 scale ^3^.

**6. Video camera monitoring and electroencephalographic recording**

Video camera monitoring (12 h/day) for spontaneous recurrent seizures (SRS) in the epileptic rats was performed. SRS was scored according to a Racine's scale assessment ^4^. Racine's scale: level 0, no epileptic seizure; level I, facial myoclonus, piloerection and scratch climb; level II, the behavior of level I together with head nodding; level III, the behavior of level II together with forelimb clonus; level IV, the behavior of level III together with hindlimb clonus or tetanus; level V, the behavior of level IV together with astasia and tumble in succession. Only the frequency of SRS in the stage IV and V animals was recorded and analyzed. For electroencephalographic (EEG) recording, 2 rats of every group were used. Three stainless steel screws were implanted at 30 days after SE, two placed over bilateral frontoparietal cortex as recording electrodes and the third over the right occipital cortex as a reference electrode. The EEG signals were recorded using a NeuroTop NT9200 EEG ampliﬁer and EEG software (Chinese Science Instrument Company, Beijing, China). The EEG was observed by a professional electrophysiologist.

**7. Morris water maze**

The rats were subjected to the test for five consecutive days. On the first day, the rats were given six trials to find the hidden platform. The rats that failed to locate the platform within 120 s were placed on the platform for 10 s for reinforcement. On the fifth day, the platform was removed and the rats received one 60 s swim probe trial. The latency to reach the platform during the training period, the total distance traveled and the frequency of platform crossing were recorded using a video cassette recorder and an image analysis system (Chinese Academy of Sciences, China).

**8. Cytoplasmic and nuclear RNA isolation**

The cytoplasmic and nuclear RNA of the HT22 cells was isolated and purified using NE-PER Nuclear and Cytoplasmic Extraction Reagents (#78833, Pierce, Rockford, IL) according to the manufacturer’s instructions. The relative level of H19 expression in each fraction was then detected by qPCR.

**9. Dual-luciferase reporter assays**

The luciferase reporter plasmid pmiR-RB-REPORT™ (RiboBio, Guangzhou, China) encoding both renialla luciferase (hRluc) and the control firefly luciferase (hluc+) was used for all assays. The 3′- UTR sequences of Stat3, c-Myc, and full length of H19 were constructed into a pmiR-RB-REPORTTM vector and co-transfected with miRNA let-7b into 293T cells. The relative luciferase activity was normalized to renilla luciferase activity 24 h after transfection.

**References**

1. Han CL, Ge M, Liu YP, et al. Long non-coding RNA H19 contributes to apoptosis of hippocampal neurons by inhibiting let-7b in a rat model of temporal lobe epilepsy. *Cell Death Dis.* 2018;9(6):617.

2. Wu X, Sun J, Zhang X, et al. Epigenetic Signature of Chronic Cerebral Hypoperfusion and Beneficial Effects of S-adenosylmethionine in Rats. *Molecular Neurobiology.* 2014;50(3):839-851.

3. Holmes GL, Sarkisian M, Ben-Ari Y, Chevassus-Au-Louis N. Mossy fiber sprouting after recurrent seizures during early development in rats. *J Comp Neurol.* 1999;404(4):537-553.

4. Racine RJ. Modification of seizure activity by electrical stimulation. II. Motor seizure. *Electroencephalography and clinical neurophysiology.* 1972;32(3):281-294.
